# Promoting activity, independence and stability in early dementia and mild cognitive impairment (PrAISED): development of an intervention for people with mild cognitive impairment and dementia

**DOI:** 10.1177/0269215518758149

**Published:** 2018-02-13

**Authors:** Vicky Booth, Rowan H Harwood, Victoria Hood-Moore, Trevor Bramley, Jennie E Hancox, Kate Robertson, Judith Hall, Veronika Van Der Wardt, Pip A Logan

**Affiliations:** 1 University of Nottingham, Nottingham, UK; 2 Nottingham University Hospitals NHS Trust, Nottingham, UK; 3 Nottinghamshire Healthcare NHS Foundation Trust, Nottingham, UK

**Keywords:** Dementia, falls, rehabilitation interventions, activities of daily living, physical activity

## Abstract

This series of articles for rehabilitation in practice aims to cover a knowledge element of the rehabilitation medicine curriculum. Nevertheless, they are intended to be of interest to a multidisciplinary audience. The competency addressed in this article is an understanding of how to develop an intervention for people with mild cognitive impairment and dementia to promote their independence, stability, and physical activity.

**Introduction:**

Older adults with dementia are at a high risk of falls. Standard interventions have not been shown to be effective in this patient population potentially due to poor consideration of dementia-specific risk factors. An intervention is required that addresses the particular needs of older people with dementia in a community setting.

**Methods:**

We followed guidelines for the development of an intervention, which recommend a structured approach considering theory, evidence and practical issues. The process used 15 information sources. Data from literature reviews, clinician workshops, expert opinion meetings, patient-relative interviews, focus groups with people with dementia and clinicians, a cross-sectional survey of risk factors, a pre-post intervention study and case studies were included. Data were synthesized using triangulation to produce an intervention suitable for feasibility testing. Practical consideration of how an intervention could be delivered and implemented were considered from the outset.

**Results:**

Elements of the intervention included individually tailored, dementia-appropriate, balance, strength and dual-task exercises, functional training, and activities aimed at improving environmental access, delivered using a motivational approach to support adherence and long-term continuation of activity. We focussed on promoting safe activity rather than risk or prevention of falls.

**Conclusion:**

We used a systematic process to develop a dementia-specific intervention to promote activity and independence while reducing falls risk in older adults with mild dementia.

## Introduction

Dementia is a global and irreversible loss of cognitive functions accompanied by a variety of neuropsychiatric symptoms and a reduced ability to perform activities of daily living.^1^ Dementia has many physical manifestations. Effects on gait and balance are seen early in the course of the disease. ‘Mild cognitive impairment’ is often a precursor to dementia, with measurable cognitive decline but no impact on daily activities. People with mild cognitive impairment present with many of the same neuropsychological and gait deficits seen in established dementia and have double the risk of falling compared with age-matched, cognitively intact, individuals. Between 60% and 80% of people with dementia fall each year.^2–4^ The impact on the individual and their support network, and associated health and social care costs, make falls a major public health issue.^5^ Dementia is progressive and causes increasing difficulty in undertaking daily activities due to failing abilities, lack of opportunity, lack of knowledge about how to adapt tasks, and fears about safety and falls.

Falls prevention interventions in older adults are well established^6^ and well evidenced.^7,8^ Despite the identification of cognitive impairment as a falls risk factor,^3,6^ interventions specifically designed for individuals with cognitive impairment are lacking. Standard interventions may be effective for people with dementia, but dementia-specific problems also require consideration, including memory loss, poor executive function, difficulties initiating activities, and increased reliance and burden on carers in performing activities. Some studies suggest that moderate- to high-intensity exercise-based training is effective at improving cognitive function,^9,10^ functional ability^11^ and reducing the risk of falls.^11,12^ Interventions have included exercise,^13^ compensatory and behavioural strategies,^14^ general physical activity,^9^ tailored strength and balance programmes^11,15^ and multicomponent packages.^12^

Current evidence does not, however, support a definitive intervention for people with dementia. There is no agreement on the components required to improve physical activity, manage risk or strategies to achieve long-term adherence. This article describes the process of developing an intervention using formal models of intervention development.

## Method

### Aim

To develop a multicomponent intervention that was dementia-specific, theoretically considered, evidence-based and feasible for people with mild dementia and mild cognitive impairment.

### Design

Intervention development was guided by the Medical Research Council guidelines for complex interventions. In the development phase, the guidance recommends identification of existing evidence, development of theoretical understanding and modelling of processes and outcomes.^16^ These categories are reflected in other frameworks, such as ‘6SQuiD’, which provided additional steps and processes.^17^ The intervention was developed through ‘coproduction’, with contributions from stakeholders including patients, carers, practitioners and policy-makers.^18^

Ethical approval was given by the National Health Service Health Research Authority for survey, interviews, focus groups and modelling components (NRES: 120966/13/EM/0161).

### Synthesis

A total of 15 component studies were used to develop the intervention. These included literature reviews,^19–24^ clinician workshops, expert opinion meetings, patient-relative interviews,^25^ focus groups with people with dementia and clinicians, a cross-sectional survey of risk factors,^26^ a pre-post ‘modelling’ intervention study and case studies. Each evidence source identified key findings. Data were synthesized using a triangulation matrix (Table 1)^27,28^ and reviewed through a series of meetings with the research management group that included clinical academic researchers, patient carer and public involvement (PCPI) representatives, and clinicians (geriatricians, psychologists, occupational therapists, physiotherapists, community nursing). Intervention components were specified according to the Template for Intervention Description and Replication (TIDieR) checklist and guide.^29^

**Table 1.** Intervention development studies’ summary of findings in relation to Medical Research Council guidance categories.

MRC category	Research method/study	Summary of findings
Evidence	Survey of people with mild dementia (*n* = 69 participants)^26^	Population is at marked risk of falls Specific neuropsychological risk factors are common Potential outcome measurements identified
	Review of gait and executive function (*n* = 11 studies)^23^	Association between poor executive function, gait and falls Potential for gait re-education and training
	Review of falls interventions in people with dementia (*n* = 7 reviews)^21^	Standard falls interventions inconsistently effective, but poorly researched Standard falls interventions identified
	Review of motivational strategies (*n* = 28 papers)^24^	‘Support’ identified as a mechanism for adherence Potential for individualized tailoring or group settings
	Review of dual-task training interventions in people with dementia (*n* = 8 studies)^20^	Dual-tasking theory refined Potential dual-tasking interventions identified Dual-task training may improve function
	Review of functional activities therapy in people with dementia (*n* = 14 papers)^22^	Potential components of therapy identified Potential assessments identified Adaptive and relearning components identified
	Workshops with clinicians and experts in field (*n* = 20 clinicians)	Components of strength and balance exercises identified Stage of dementia influences choice of restorative or compensatory approach Environmental components for minimizing risk, adaptations, insight and education identified Treatment programme should include goal-setting to tailor intervention for individual Practical components for delivery and materials
	Interviews with people with dementia and their relatives (*n* = 20 dyads)^25^	People with mild dementia normalized the occurrence of a fall and attributed them to the environment, unfamiliar surroundings, misjudgement or chance; ongoing risk poorly recognized Barriers and facilitators to falls interventions identified Exercise was acceptable in principle Maintaining activity and independence were key goals
Theory	Expert advice on functional activities and dual-tasking (*n* = 2 meetings)	Dual-task assessment and intervention rationale, procedures and tailoring according to participant ability Recommendations compared for those with and without cognitive impairment
	Realist review of exercise in dementia (*n* = 35 papers)^19^	Identified mechanisms underpinning impact of exercise Exercise engages physiological-responses involving motor, postural, gait and cognitive processes Human support is important for people with cognitive impairment Both the individual and the supporter for exercise needs to perceive a benefit (not necessarily related to falls) Exploration of intervention ‘dose’
	Synthesis of adherence and motivation (*n* = 3 theories)	Identification of a range of theoretically derived and evidence-based behaviour change approaches and practical strategies (e.g. goal-setting, prompts/cues, graded tasks, habit formation) Self-determination theory selected to inform the design and delivery style of the intervention
Modelling	Interviews with clinicians (*n* = 19 clinicians)	Recognize close association between falls and dementia Services are limited by what they are commissioned to provide Current model of falls prevention delivery poorly suited to people with dementia therefore need to extend services
	Pre-post exercise intervention study with people with mild dementia (*n* = 10 participants)	Intervention deliverable, feasible and acceptable in population Dual-task and exercise concept can be practically implemented in people with mild dementia Measurable changes in intermediate outcomes
	Case studies (*n* = 2 participants)	Functional-component of intervention deliverable, feasible and acceptable in people with insight into mild dementia Delivery and content refined
	Focus groups with clinicians and people with dementia (*n* = 13 participants)	Motivation support can come from self, others, gadgets and be included in the design of the intervention Tailoring the motivation support to the individual’s needs is key to continued engagement in exercises and activities

MRC: Medical Research Council.

## Results

The rationale for intervention components (‘why’) and equipment or procedures used (‘what’) were well populated from the 15 studies.

### Intervention description (TIDieR checklist)

#### Name

Promoting activity, independence and stability in early dementia and mild cognitive impairment (PrAISED).

#### Why: rationale, theory and goals

Older adults with mild dementia have a high risk of falls. This was evidenced by literature^2,15^ and through a bespoke cross-sectional survey of 69 adults (mean age 81 years) with mild dementia or mild cognitive impairment (Montreal Cognitive Assessment = 15–25/30, median = 21). One-third had fallen in the previous six months and risk of falls was high (mean score = 2.5) according to the Physiological Profile Assessment.^30^ Falls risk was significantly associated with spatial memory abilities and poor inhibition of a pre-potent response.^26^ Gait pattern and balance were impaired and significantly associated with global cognition.^31^

As well as standard falls risk factors associated with age and comorbidities, there are dementia-specific risk factors that need addressing.^2^ A systematic literature review highlighted that poor executive function was associated with increased falls risk and decline in gait speed.^23^

A review of falls prevention for people living with dementia interventions identified seven systematic reviews, in which exercise and multicomponent programmes were most commonly reported. Evidence on efficacy on falls prevention was inconsistent.^21^ Current interventions do not account for dementia-specific risk factors.

Semi-structured interviews with people living with dementia and their relatives revealed that they were resistant to the idea of intervention to reduce falls.^25^ Few saw themselves as likely to fall, despite many having experienced falls, or expressing concern about falls.^25^ The occurrence of a fall was ‘normalised’ and rationalized in terms of environmental hazards, unfamiliar surroundings, misjudgement (‘own silly fault’) or chance. Exercise as an intervention was acceptable in principle, as were equipment and adaptations. However, a pure exercise programme alone was unlikely to be followed. Instead, any intervention should be directed at goals seen as important, including maintaining activity, independence and social engagement.

Activity and independence may be best maintained, and falls risk reduced, by intervening at an early stage.^32–35^ This rationale was identified within the literature and confirmed through discussions with international experts in the field.

Exercise at the correct intensity and duration can reduce falls risk in older adults.^36^ A systems theory approach to balance control and falls risk assessment and prevention has been proposed.^30^ Adding dual-task training (aiming to reduce the impact of executive dysfunction) may enhance standard strength and balance exercises. A meta-analysis of eight studies reported a wide variety of such programmes and produced a list of dual-tasking exercises.^20^ Pooled data identified a statistically significant improvement in falls-related outcomes of balance and gait speed following the intervention.

Functional activity assessment, adaptation and relearning can enhance independence and maintenance of activity, as well as identifying and addressing falls risk factors. In a narrative review of 14 studies, the key characteristics of a functional intervention for people with dementia were evidenced, including a number of formal assessments.^22^ Adaptive or compensatory strategies, cognitive approaches, and relearning approaches were featured but had different strengths and limitations. A combination of environmental adaptation and cognitive rehabilitation approaches had potential to maximize outcomes. Experienced researchers and clinicians in the field of falls prevention and dementia participated in two workshops (*n* = 20 participants), where it was reported that a compensatory or functional approach was often adopted in practice when treating patients with dementia and poor balance.

A key challenge with exercise interventions is how to achieve adherence. The PrAISED intervention is underpinned by motivational theory and engagement strategies to optimize uptake and adherence.^24^ Data from multiple sources (literature reviews, interviews, focus groups, PCPI consultation and group discussion) were mapped to relevant behaviour change frameworks (‘capability’, ‘opportunity’, ‘motivation’ and ‘behaviour’ [COM-B]^37^), theoretical domains^38^ and a range of techniques and practical strategies (e.g. goal-setting, prompts/cues, graded tasks, habit formation) to support participants’ motivation and adherence. Following a review of behaviour change models, self-determination theory^39^ was used as the main psychological theory informing the design and delivery of the intervention. This has parallels with person-centred dementia care, including both content and the style of delivery (i.e. need-supportive motivation communication strategies such as taking time to understand the participant and acknowledging the participant’s feelings^40^).

#### What – materials: provider, participant and equipment

Materials provided to participants were trialled during a ‘modelling’ study in which 10 people with mild dementia undertook a supervised, six-week balance-challenging exercise and dual-task training intervention. Printed instructions were left in the participant’s home and included the following:

- Visual and written description of strength and balance exercises, dual-task exercises and functional activities;
- A checklist to encourage discussion on interests;^41^
- Weekly activities and plan documentation;
- Goals to achieve during the intervention;
- Information on community-based activities;
- Environmental adaption or risk enablement documentation;
- A record of the visits.

Equipment for intervention sessions was trialled during this study and included therapeutic balls, variable cuff weights, household items such as a cup or glass, steps or stairs within the home, and functional activities items such as cooking materials, clothing or other household items.

#### What – procedure: provider training, assessment and intervention session

The intervention goes beyond standard proactive clinical practice and requires specific training. Clinician training was designed for registered therapists and therapy support workers. Core elements included a series of teaching days, paper copy of an intervention manual, specific training on motivation and adherence, electronic access to all intervention content, ongoing online peer and expert support, and face-to-face support from the intervention developers.

The assessment procedure was refined during the cross-sectional survey and a case study involving two carer representatives. Standardized assessments include falls risk assessment (Guide-to-Action,^42^ postural blood pressure), functional assessment (informed by the Assessment of Motor and Process Skills^43^), physical assessment (muscle strength, Berg Balance Scale,^44^ Timed Up and Go-Dual Task) and goal-setting.

The structure of the intervention sessions was tested during the ‘modelling’ study and involved feedback from the participant regarding previous session or daily activities; functional activity (repetition and practice of functional activities); individually set balance, strength and dual-task exercises (based on the Otago programme^45^); engagement with community such as walking; and documentation of the session.

#### Who provides: therapists delivering intervention

Intervention providers included registered Physiotherapists and Occupational Therapists with support from unregistered assistants (Rehabilitation Support Workers). Bramley et al.^22^ identified functional interventions were best delivered by registered clinicians, which was confirmed through the clinician workshops. Experience of working with either older people who fall or people with dementia was also identified from the clinician workshops as valuable to aid assessment and treatment planning.

#### How: method of delivery

The intervention was delivered face-to-face, in a one-to-one method during the ‘modelling’ study. To enhance adherence, the delivery method has been informed by self-determination theory.^39^ This involves need-supportive and motivation communication strategies (e.g. taking time to understand the participant, acknowledging participant’s feelings^40^), as well as motivational strategies, such as goal-setting, prompts/cues, graded tasks and habit formation. These strategies will be further refined during feasibility testing.

#### Where: place of delivery

The intervention sessions are completed in the participant’s home. Both home and group settings for delivery were considered and trialled during the ‘modelling’ study. Home delivered sessions promoted tailoring, progression and adaptation of the intervention more than a group setting, in keeping with other studies where functional outcomes and patient preference favoured a home setting.^11,46^ Delivery in a (small) group invariably required one-to-one support in practice. Carers or family members were invited to attend and take part in sessions.

#### When and how much

Evidence is not yet available that determines the amount of exercise that people with dementia need to accomplish to reduce falls; therefore, guidance is taken from research conducted in other older people.^36^ The intervention sessions aim to facilitate physical activity of 60 minutes duration, three times a week, some directly supervised. The programme is designed to be continued for a minimum of six months, with encouragement to continue indefinitely, or as long as physically able. To this end, supervision is tailored to abilities and available support is tapered to promote self-directed or carer-supported exercise, and community facilities or groups are signposted.

#### Tailoring the intervention

A core principle of the intervention is that it is individually tailored to the abilities, comorbidities, interests and goals of the participant. Tailoring the interventions to overcome barriers of content specificity (i.e. adapting the exercises and activities), delivery, motivation and adherence arose from patient-carer and clinician interview studies and from expert advice. Goal-setting and an interest checklist are used to tailor the intervention on patient interests which has been established as a motivational factor from multiple sources (motivational systematic review, clinician workshops and ‘modelling’ study). Progression of effort was an important aspect identified from the literature^47^ and ‘modelling’ study and is achieved through increasing the number of repetitions, resistance or time completing each exercise, and reducing base of support for the balance exercises (e.g. removing touch support or narrowing base of support).

#### Modifications

The intervention is being tested in a feasibility study, which will be the basis for further refinement.

#### How well: planned and actual

These will be assessed and document in a feasibility study.

## Discussion

### Summary of findings

An intervention, called PrAISED, has been developed to support people with early stage dementia to remain independent for longer and to reduce fall risk using a systematic approach to design and synthesizing key findings from 15 study components using a triangulation matrix. Data sources recommend that participants require a combination of physical exercises, functional activities, and psychological and cognitive techniques. The PrAISED intervention has been designed to be delivered to community-dwelling populations in the first instance by physiotherapists and occupational therapists with individuals then self-managing the programme.

### Strengths and limitations

A mixed methods design was employed to develop this intervention. Quantitative and qualitative data were rigorously collected, analysed according to convention and synthesized with equal value. Mixed methods research is well established in health research^48^ and the rationale for its use was sound. The approaches used were complementary to each other, providing incremental progression from one study and research question to another and corroborating findings identified separately to enhance overall validity.^48^

The multidisciplinary and professional range in the study steering group was helpful. Groups are complex and dynamic, and events and decision-making processes can be difficult to report. Various theories are associated with group-decision making, such as Groupthink^49,50^ and social-identity theory.^51^ Reflexivity was therefore important to remain open and objective.

PCPI involvement has been utilized at all stages of the intervention development process, providing a grounding in lived experience for the development process.

All categories of intervention development described by the Medical Research Council (MRC) framework have been included. Formal criteria from TIDieR and the Criteria for Reporting the Development and Evaluation of Complex Interventions (CReDECI) guidelines were followed.^29,52^ The intervention has been described according to the TIDieR guidelines (Supplementary material 1).

Throughout the development process, the component studies have been published and presented at regional, national and international conferences. These dissemination events have allowed the work to be peer-reviewed and challenged, ensuring relevance to the field of study and quality of reporting.

The most significant limitation is the nature of the intervention development process. The involvement of clinicians in the process has ensured relevance to practice, but it is challenging to describe and document the mix of clinical knowledge, experience and intuition. Defining and reporting all findings, positive and negative, is difficult. The amount of work and depth of detail in the qualitative elements are summarized into simple and restricted sentences that potentially do not reflect the nuanced findings. Publication bias may favour those aspects of the process that produced positive results. Transparency in the development process is important.

### Context and comparison

Physical activity and multicomponent interventions have been suggested to reduce falls^12^ and improve function^11^ and gait.^53–55^ These studies have resulted from similar developmental work.

Has this process delivered an intervention that is ready for further testing? The Medical Research Council’s complex intervention guidelines are broad, providing a general framework that covers all aspects of development from theory identification to evaluation by randomized controlled trial and implementation.^56^ However, there is sparse detail, especially in critical initial stages. In general, few intervention development studies are published.^57^ There is little certainty on when an intervention has been developed ‘enough’ to be optimally effective, implemented or tested by randomized controlled trial.

Overall, the intervention development process of this study was successful. The intervention is ready for practical implementation and further refinement through experimental study (feasibility trial). Explanation as to how it worked, why it might not have worked, and how it might be further developed or improved will be through a process evaluation and testing in the feasibility randomized controlled trial.

### Implementation and recommendations

Currently, the intervention is not suitable to be implemented into clinical practice as its efficacy has not been determined and the training of intervention providers needs to be evaluated. This work is part of an ongoing NIHR-funded Programme Grant (RP-PG-0614-20007).

**Clinical messages**

- Developing a new intervention should be a considered, planned and detailed process which is clearly documented.
- People with dementia have a higher risk of falling and require individualized intervention programmes to reduce this risk.
- A dementia-specific intervention (PrAISED) has been developed which is suitable for feasibility testing.

## Supplementary Material

Supplementary material
