# Supplementary material for: Promoting activity, independence and stability in early dementia and mild cognitive impairment (PrAISED): development of an intervention for people with mild cognitive impairment and dementia
Source: Clin Rehabil. 2018 Feb 13;32(7):855–64. doi: 10.1177/0269215518758149 (PMC6039861; doi:10.1177/0269215518758149)
Supplement: Supplementary material [file cre-2017-6411-File005.pdf]

Supplementary Material 1: Table to describe the PrAISED intervention according to the TIDieR checklist.

TIDieR checklist item				Intervention item and brief description
**1.**	**Name**			An intervention to promote activity, independence, and stability in early dementia and mild cognitive impairment (PrAISED).
**2.**	**Why**		Rationale	Older adults with mild cognitive impairment have high risk of falls. As well as standard falls risk factors associated with age and comorbidities, dementia-specific risk factors need addressing, which current interventions do not account for persons with dementia do not consider falls risks, but value maintenance of activity and independence.
			Theory	• By intervening at an early-stage, activity and independence can be enhanced and maintained, and falls risk reduced.<br>• Exercise at the correct intensity and duration can reduce falls risk.<br>• Adding dual-task training (mitigating the effect of executive dysfunction) can enhance standard strength and balance exercises and incorporate cognitive risk factors for falls.<br>• Functional activity assessment, adaptation and relearning can enhance independence and maintenance of activity, as well as identifying and addressing falls risk.<br>• Promotion and engagement with community-outdoor environment can enhance duration of involvement in activity and encourage independence.<br>• An intervention implemented according to motivational theory and supported by engagement strategies will improve uptake and adherence.
			Goal	To promote activity and independence, and prevent falls, for older people with early dementia and mild cognitive impairment living in the community.
	**What**	**3.**	**Materials**	*Provider*: Specific training provided to therapists and support workers who undertake the intervention sessions with the participant. This involves training days, an intervention manual, a motivation manual, electronic access to intervention content, on-going online peer support, and face-to-face support from intervention developers, as required.<br>*Participants*: Intervention content printed on paper and collated into a 'home-file' folder. The content includes:<br>• Clinician contact.<br>• Strength and balance exercises (based on standard programmes i.e. Otago).<br>• Dual-task exercises.<br>• Functional activities.<br>• Interest checklist<br>• 'My week' planning form.<br>• Goal-setting sheet.<br>• Information on community-based activities.<br>• Environmental adaption or risk enablement.<br>• Visit record.<br>Equipment for intervention sessions includes: therapeutic balls, variable cuff weights, household items such as a cup or glass, steps or stairs within the home, and functional activities items such as cooking materials, clothing or other household items.
		**4.**	**Procedures**	*Provider training*: Group training is provided to the therapists and support workers conducting intervention sessions. This includes intervention rationale, aims, assessments and content.<br>*Assessment procedure*: Falls risk assessment (Guide to Action, blood pressure), functional assessment (informed by Assessment of Motor and Process Skills), physical assessment (muscle strength, Berg Balance Scale, Timed Up and Go-Dual Task), and goal-setting.

Supplementary Material 1: Table to describe the PrAISED intervention according to the TIDieR checklist.

			Intervention sessions: feedback from the participant regarding previous session or daily activities; functional activity (thinking-doing activities); individually set balance, strength, and dual-task exercises; engagement with community such as walking.
**5.**	**Who**		Intervention providers are registered Physiotherapists (PT) and Occupational Therapists (OT) with support from unregistered Rehabilitation Support Workers (RSW). Providers should have experience working with either older people who fall or people with dementia. All providers should participate in the training programme (Section 3) before conducting intervention sessions.
**6.**	**How**		The intervention is delivered face-to-face. Carers or family members are invited to attend sessions.<br>Motivational strategies (e.g., goal-setting, prompts/cues, graded tasks, habit formation) and a need-supportive communication style are used to enhance adherence and uptake.
**7.**	**Where**		The intervention sessions are completed in the participant's home. Where functional or community activities require, intervention sessions may be conducted in the community, according to the participant's goals and ability.
**8.**	**When**		Intervention sessions will facilitate physical activity three times a week, for one year duration in the first instance. Some intervention sessions are supervised, the amount of supervision is tailored according to prior activity levels, ability, co-morbidity, carer availability to help, and goals.<br>Each supervised session lasts approximately 60 minutes. Where possible, the day and time of supervised intervention sessions are maintained, and incorporate at least a 48 hour interval between sessions.
**9.**	**Tailoring**		The programme will be tailored according to abilities, comorbidities, interests and goals of the individual participant. These will be established during the initial session of the intervention and monitored by the registered therapists and RSW. The programme will be progressed to maintain an achievable challenge for each participant. Progression will be achieved through:<br>• Increasing the number of repetitions or time completing each exercise.<br>• The resistance for the strength exercises.<br>• Reducing base of support for balance exercises (for example removing touch support or narrowing base of support).<br>• Increasing the difficulty of the dual-task component.<br>• Reducing the number of prompts or adaptions provided in the functional task.<br>• Increasing the complexity of the functional task.<br>• Reducing the support given by the intervention providers.
**10.**	**Modifications**		n/a
	**How well**	**11. Planned**	Intervention adherence will be measured by;<br>• Self-report activity calendars and pedometers.<br>Intervention fidelity will be measured by;<br>• Self-report of intervention delivery
		**12. Actual**	n/a
